# Dietary fat and breast cancer risk revisited: a meta-analysis of the published literature

**DOI:** 10.1038/sj.bjc.6601314

**Published:** 2003-10-28

**Authors:** N F Boyd, J Stone, K N Vogt, B S Connelly, L J Martin, S Minkin

**Affiliations:** 1 Division of Epidemiology and Statistics, Ontario Cancer Institute, 610 University Avenue, Toronto, Ontario, Canada M5G 1K9

**Keywords:** diet, fat, breast cancer risk, meta-analysis

## Abstract

Animal experiments and human ecological studies suggest that dietary fat intake is associated with a risk of breast cancer, but individual-based studies have given contradictory results. We have carried out a meta-analysis of this association to include all papers published up to July 2003. Case–control and cohort studies that examined the association of dietary fat, or fat-containing foods, with risk of breast cancer were identified. A total of 45 risk estimates for total fat intake were obtained. Descriptive data from each study were extracted with an estimate of relative risk and its associated 95% confidence interval (CI), and were analysed using the random effects model of DerSimonian and Laird. The summary relative risk, comparing the highest and lowest levels of intake of total fat, was 1.13 (95% CI: 1.03–1.25). Cohort studies (*N*=14) had a summary relative risk of 1.11 (95% CI: 0.99–1.25) and case–control studies (*N*=31) had a relative risk of 1.14 (95% CI 0.99–1.32). Significant summary relative risks were also found for saturated fat (RR, 1.19; 95% CI: 1.06–1.35) and meat intake (RR, 1.17; 95% CI 1.06–1.29). Combined estimates of risk for total and saturated fat intake, and for meat intake, all indicate an association between higher intakes and an increased risk of breast cancer. Case–control and cohort studies gave similar results.

Breast cancer is the leading cause of cancer death, and the most frequently diagnosed cancer in women worldwide (Lacey *et al*, 2002). Large differences in rates of the disease exist between countries, with higher rates in North America and Western Europe, and lower rates in Asia and South America (Lacey *et al*, 2002). These differences are likely to be due to environmental rather than genetic factors. The rates of breast cancer change in migrants from low- to high-risk countries, who eventually acquire the rates of their adopted country (Ziegler *et al*, 1993; Pike *et al*, 2002). Menstrual and reproductive risk factors for breast cancer do not appear to account for these differences in rates (Wu *et al*, 1996).

The differences in dietary practices between countries are well established, and could contribute to the differences in breast cancer risk. Support for an influence of dietary fat on breast cancer rates comes from its effect on mammary carcinogenesis in animals, and human ecological data.

Two major meta-analyses, combining results from over 140 studies examining the relationship between dietary fat and breast cancer risk in rats and mice, show dietary fat to be a promoter of mammary carcinogenesis (Fay *et al*, 1997). This effect is independent of the effects of caloric intake (Freedman *et al*, 1990). Human ecological studies show a strong correlation (0.7 or more) between dietary fat intake, estimated from national food balance data, and incidence and mortality of breast cancer worldwide. (Prentice and Sheppard, 1990).

However, case–control and cohort studies that have examined the relationship between dietary fat and breast cancer risk in humans have given inconclusive results. In 1993, we conducted a meta-analysis of the 23 studies then published that gave risk estimates for the total dietary fat, type of fat or for fat-containing foods (Boyd *et al*, 1993). The number of published primary research papers on this issue has since then more than doubled. The present analysis updates and expands our earlier meta-analysis to include all studies on this relationship published since 1993.

## METHODS

### Assembly of literature

Case–control and cohort studies for inclusion in the analysis were identified by searching the MEDLINE and PUBMED databases for literature on the intake of fat, fat subtypes and fat-containing foods, and breast cancer risk over the period from January 1966 to July 2003. Reference lists of review articles and primary studies were also searched for additional relevant literature.

A total of 46 risk estimates for total fat intake were obtained from the 45 independent studies included in the meta-analysis (see Table 1

**Table 1:** Selected characteristics of (A) case–control studies: total fat and (B) cohort studies: total fat

**Author**	**Country**	**No. of cases**	**No. of controls**	**Type of controls**	**Dietary assessment**	**Partition**	**RR total fat**	**Quality score**
*(A)*								
Challier (1998)	France	345	345	Centre	Diet history^a^^,^^b^	Quintile	1.71 (0.77,3.76)	6/7
De stefani *et al* (1998)	Uruguay	365	397	Hospital	Food freq^b^^,^^c^	Quartile	1.53 (0.89,2.62)	6/7
Ewertz and Gill (1990)	Denmark	1474	1322	Population	Food freq^b^^,^^c^	Quartile	1.45 (1.17,1.80)	3/7
Franceschi *et al* (1996)	Italy	2569	2588	Hospital	Food freq^b^^,^^c^	Quintile	0.81 (0.63,0.99)	6/7
Graham *et al* (1982)	USA	1803	917	Hospital	Food freq^b^^,^^c^	Quartile	0.9 (0.5,1.5)	5/7
Graham *et al* (1991)	USA	439	494	Population	Food freq^b^^,^^c^	Quartile	0.93 (0.63,1.38)	5/7
Hirohata *et al* (1985)	Japan	212	424	Hospital and neighbourhood	Diet history^b^	Quartile	1.01 (0.60,1.71)	3/7
Hirohata *et al* (1987)	Hawaii							
Japanese		J 183	183	Neighbourhood	Diet history^b^	Quartile	1.5 (0.8,2.9)	5/7
Caucasian		C 161	161	Neighbourhood	Diet history^b^	Quartile	1.3 (0.6,2.6)	
Holmberg *et al* (1994)	Sweden	265	432	Population	Food freq^b^^,^^c^	Quartile	1.3 (not given)	6/7
Ingram *et al* (1991)	Australia	99	209	Population	Food freq^b^^,^^c^	Median of fat intake	1.4 (0.8,2.5)	5/7
Katsouyanni *et al* (1988)	Greece	120	120	Hospital	Food freq^c^	90th *vs* 10th percentiles	1.36 (0.69,2.67)	4/7
Katsouyanni *et al* (1994)	Greece	820	1546	Hospital	Food freq^c^	Quintile	0.94 (0.85,1.05)	5/7
Landa *et al* (1994)	Spain	100	100	Hospital	Food freq^c^	Tertile	0.29 (0.1,0.7)	4/7
Lee *et al* (1991)	Singapore	200	420	Hospital	Food freq^c^	Tertile	0.75 (0.41,1.36)	4/7
Levi *et al* (1993)	Switzerland	107	318	Hospital	Food freq^c^	Tertile	1.53 (0.86,2.71)	5/7
Mannisto *et al* (1999)	Finland	310	454	Population	Food freq^a^^–^c	Quintile	0.7 (0.3,1.6)	7/7
Martin-Moreno *et al* (1994)	Spain	762	988	Population	Food freq^a^^–^c	Quartile	0.98 (0.74,1.29)	7/7
Miller *et al* (1978)	Canada	400	400	Population	Diet history^b^	Tertile	1.6 (0.9,3.0)	5/7
Nunez *et al* (1996)^d^	Spain	139	136	Hospital	Diet history	Tertile	2.04 (0.84,4.99)	4/7
Potischman *et al* (1998)	USA	1647	1501	Population	Food freq^c^	Quartile	1.00 (0.8,1.2)	4/7
Pryor *et al* (1989)	USA	172	190	Population	Food freq^b^^,^^c^	Quartile	0.7 (0.3,1.5)	5/7
Richardson *et al* (1991)	France	409	515	Hospital	Diet history	Tertile	1.6 (1.1,2.2)	6/7
Rohan *et al* (1988)	Australia	451	451	Population	Food freq^a^^–^c	Quintile	0.9 (0.59,1.38)	6/7
Shun-Zhang *et al* (1990)	China	186	372	Population & hospital	Diet history^b^	Quintile	1.67 (1.01,2.05)	6/7
Toniolo *et al* (1989)	Italy	250	499	Population	Diet history^b^	Quartile	1.8 (0.98,3.29)	6/7
Trichopoulou *et al* (1995)	Greece	820	1548	Hospital	Food freq^b^^,^^c^	Quintile	1.01 (0.94,1.08)	6/7
Van't Veer *et al* (1990, 1991)	Netherlands	133	289	Population	Diet history^b^	Per 24 g fat	1.54 (1.06,2.22)	6/7
Wakai *et al* (2000)	Indonesia	226	452	Hospital	Food freq^b^^,^^c^	Quartile	5.43 (2.14,13.77)	6/7
Witte *et al* (1997)	USA/Canada	140	222	Sisters	Food freq^a^^–^c	Quartile	0.4 (0.2,0.8)	6/7
Yuan *et al* (1995)	China	834	834	Population	Food freq^c^	Per 90 g fat	1.2 (0.7,2.0)	5/7
Zaridze *et al* (1991)	Moscow	139	139	Clinic	Food freq^c^	Quartile	0.52 (0.04, 6.99)	5/7

Total cases	16 280							
Total controls	18 966							

*(B)*								
Bingham *et al* (2003)	UK	168	672	Population	Diet history^b^	Quintile	1.31 (0.65,2.64)	6/6
Cho (2003)	USA	714	90 655	Population	Food freq^a^^–^c	Quintile	1.25 (0.98, 1.59)	6/6
Gaard *et al* (1995)	Norway	248	24 897	Population	Food freq^a^^–^c	Quartile	1.25 (0.86,1.81)	6/6
Graham *et al* (1992)	USA	359	18 586	Population	Food freq^a^^–^c	Quintile	0.99 (0.69,1.41)	6/6
Holmes *et al* (1999)	USA	2956	88 795	Population	Food freq^a^^–^c	Quartile	0.97 (0.94,1.00)	5/6
Howe *et al* (1991a, b)	Canada	519	56 837^e^	Population	Diet history^a^^,^^b^	Quartile	1.35 (1.00,1.82)	6/6
Jones *et al* (1987)	USA	99	5495	Population	24 h recall	Quartile	0.34 (0.16,0.73)	3/6
Knekt *et al* (1990)	Finland	54	3988	Population	Diet history^b^	Tertile	1.72 (0.61,4.82)	6/6
Kushi *et al* (1992)	USA	459	34 388	Population	Food freq^a^^–^c	Quartile	1.16 (0.87,1.55)	6/6
Thiebaut and Clavel-Chapelon (2001)^f^	France	838	65 879^g^	Population	Food freq^a^^–^c	Quartile	1.37 (0.99,1.89)	6/6
Toniolo *et al* (1994)	USA	180	14 291^h^	Population	Food freq^a^^–^c	Quintile	1.49 (0.89,2.48)	6/6
van den Brandt *et al* (1993)	Netherlands	471	62 573^i^	Population	Food freq^a^^–^c	Quintile	1.08 (0.73,1.59)	6/6
Velie *et al* (2000)	USA	996	40 022	Population	Food freq^a^^–^c	Quintile	1.07 (0.86.1.32)	6/6
Wolk *et al* (1998)	Sweden	674	61 471	Population	Food freq^a^^–^c	Quartile	1.0 (0.76,1.32)	6/6

Total cases	8735							
Total population	568 549							

a Self-administered.

b Diet assessment method validated.

c Food Frequency Questionnaire.

d Article translated from Spanish.

e No. of controls in calculation of RR=1182.

f Article translated from French.

g No. of controls in calculation of RR=62 211.

h No. of controls in calculation of RR=829.

i No. of controls in calculation of RR=1598.

for references). Risk estimates for types of fat were also extracted from the 33 studies that provided them.

Studies were also identified that contained information regarding food groups and breast cancer risk. The three most common foods for which risk estimates were given in these studies were determined (meat, milk and cheese) and used in the present meta-analysis. Two studies, which defined food groups in a manner that could not be adapted to this analysis, were excluded (Katsouyanni *et al*, 1986; Lubin *et al*, 1986). Risk estimates pertaining to the intake of these foods were obtained from a total of 36 papers (see Table 2

**Table 2:** Selected characteristics of (A) case–control studies: food and (B) cohort studies: food

**Author**	**Country**	**No. of cases**	**No. of controls**	**Type of controls**	**Dietary assessment**	**Food**	**No of categories^a^**	**RR^i^**	**CI**	**Quality score**
*(A)*										
Ambrosone *et al* (1998)	USA	740	810	Population	Food freq^b^	Meat^c^	4	0.92	(0.25, 3.32)	5/7
De stefani *et al* (1997)	Uruguay	352	382	Hospital	Food freq^b^^,^^d^	Meat	4	2.26	(1.24, 4.12)	5/7
Ewertz and Gill (1990)	Denmark	1474	1322	Population	Food freq^b^^,^^d^	Meat	6	0.94	(0.63, 1.40)	3/7
						Milk	5	1.45	(1.02, 2.07)	5/7
Franceschi *et al* (1995)	Italy	2569	2588	Hospital	Food freq^b^^,^^d^	Meat	4	0.99	(0.69, 1.41)	6/7
						Milk	5	0.81	(0.67, 0.98)	
						Cheese	5	0.98	(0.81, 1.18)	

Hirohata *et al* (1987)	USA	183	183	Population	Diet history^d^	Meat	4	1.5	(0.7,3.1)	5/7
Hislop *et al* (1986)	Canada	846	862	Population	Food freq^b^^,^^e^	Meat	3	1.16	(0.90, 1.48)	3/7
						Milk	3	1.55	(1.18, 2.05)	

Holmberg *et al* (1994)	Sweden	265	432	Population	Food freq^b^^,^^d^	Meat	8	0.8	(0.5, 1.2)	6/7
Ingram *et al* (1991)	Australia	99	209	Population	Food freq^b^	Meat	2	1.6	(0.9, 2.8)	3/7
						Milk	2	0.9	(0.5, 1.6)	

Kato *et al* (1992)	Japan	908	908	Hospital	Unspecified	Meat	3	0.75	(0.60, 0.94)	2/7
Landa *et al* (1994)	Spain	100	100	Hospital	Food freq^b^	Meat	3	1.21	(0.31, 4.66)	4/7
La Vecchia *et al* (1987)	Italy	1108	1281	Hospital	Food freq^b^	Meat	3	1.39	(1.12, 1.71)	4/7
Le *et al* (1986)	France	1010	1950	Hospital	Food freq^b^	Milk	3	1.8	(1.3, 2.4)	5/7
						Cheese	3	1.5	(1.0, 2.3)	

Lee *et al* (1991)	Singapore	200	420	Hospital	Food freq^b^	Meat	3	1.4	(0.77, 2.53)	4/7
Levi *et al* (1993)	Switzerland	107	318	Hospital	Food freq^b^	Meat^c^	3	1.45	(0.56, 3.72)	5/7
						Milk	3	1.15	(0.68, 1.96)	
						Cheese	3	2.99	(1.7, 5.25)	

Lubin *et al* (1981)	Canada	577	826	Population	Food freq^b^	Meat	3	1.42	(1.0, 2.0)	4/7
						Milk	4	0.77	(0.5, 1.3)	
						Cheese	3	1.11	(0.9, 1.4)	

Mannisto *et al* (1999)	Finland	310	454	Population	Food freq^b^^,^^d^^,^^e^	Meat	5	0.66	(0.12, 3.72)	7/7
						Milk	4	1.7	(0.8, 3.66)	
						Cheese	4	0.75	(0.3, 1.7)	

Matos *et al* (1991)	Argentina	196	205	Neighbourhood	Food freq^b^	Meat	3	1.4	(0.7, 2.9)	4/7
Potischman *et al* (1998)	USA	1647	1501	Population	Food freq^b^	Meat	4	1.18	(1.0, 1.5)	4/7
Richardson *et al* (1991)	France	409	515	Hospital	Diet history^b^	Meat	3	1	(0.7, 1.4)	6/7
						Cheese	3	1.4	(1.0, 1.9)	

Talamini *et al* (1984)	Italy	368	373	Hospital	Food freq	Meat	3	1.3	(0.7, 2.2)	4/7
						Milk	3	3.2	(1.85, 5.8)	

Toniolo *et al* (1989)	Italy	250	499	Population	Diet history^d^	Meat^c^	4	1.15	(0.82, 1.62)	6/7
						Milk	4	1.73	(1.16, 2.6)	
						Cheese	4	2.6	(1.7, 4.0)	

Trichopoulou *et al* (1995)	Greece	820	1548	Hospital visitors	Food freq^b^^,^^d^	Meat	5	1.07	(0.99, 1.15)	6/7
						Milk	5	1.0	(0.93, 1.08)	

Van't Veer *et al* (1989)	Netherlands	133	289	Population	Diet history	Milk	Per 225 g	0.81	(0.59, 1.12)	4/7
						Cheese	Per 60 g	0.56	(0.33, 0.95)	

Witte *et al* (1997)	USA/Canada	140	222	Population	Food freq^b^^,^^d^^,^^e^	Meat	4	0.6	(0.3, 1.3)	6/7
Wang *et al* (2000)^f^	China	2063	2063	Neighbourhood	Food freq^b^	Milk	Per 500 g	1.49	Not given	

Total cases	16 734									
Total controls	20 038									

*(B)*										
Cho (2003)	USA	714	90 655	Population	Food freq^b^^,^^e^^,^^d^	Meat^c^	5	1.11	(0.92, 1.35)	6/6
Gaard *et al* (1995)	Norway	248	25 897	Population	Food freq^b^^,^^e^^,^^d^	Meat	4	2.28	(1.29, 4.03)	6/6
						Milk	4	1.71	(0.86, 3.38)	

Gertig *et al* (1999)	USA	466	466	Population	Food freq^b^^,^^d^^,^^e^	Meat^c^	3	1.06	(0.48, 2.33)	6/7
Hirayama (1978)	Japan	139	142 857	Population	National nutrition survey	Meat	2	1.7	(0.8, 3.8)	3/6
Hjartaker *et al* (2001)	Norway	317	48 844	Population	Food freq^b^^,^^d^^,^^e^	Milk	3	0.51	(0.27, 0.96)	5/6
Kinlen (1982)	Britain	62	2813	Population	Unspecified	Meat	2	1.2	(0.8, 1.6)	2/6
Knekt *et al* (1996)	Finland	88	4697	Population	Food freq^b^^,^^d^	Milk	3	0.42	(0.24, 0.74)	4/6
						Cheese	3	1.25	(0.75, 2.08)	

Mills *et al* (1989)	USA	215	20 341	Population	Food freq	Meat^c^	3	1.11	(0.47, 2.66)	4/6
						Milk	3	0.94	(0.66, 1.33)	
						Cheese	3	1.43	(0.99, 2.06)	

Thiebaut and Clavel-Chapelon (2001)^g^	France	838	65 879	Population	Food freq^b^^,^^d^^,^^e^	Cheese	4	0.92	(0.74, 1.13)	6/6
Toniolo *et al* (1994)	USA	180	829	Population	Food freq^b^^,^^d^^,^^e^	Meat	5	1.44	(0.68, 3.04)	6/6
van den Brandt *et al* (1993)	Netherlands	437	62 573^h^	Population	Food freq^b^^,^^d^^,^^e^	Meat	Not given	1.23	(0.63, 2.37)	6/6
Vatten *et al* (1990)	Norway	152	14 500	Population	Food freq^b^^,^^d^	Meat	3	1.8	(1.1, 3.1)	4/6

Total cases	3783									
Total controls	476 200									

a Food Frequency Questionnaire.

b Self-administered.

c Diet assessment method validated.

d No. of categories refers to the number of categories of frequency of consumption into which the food intakes were partitioned. The RR is the highest *vs* lowest level of consumption.

e Article translated from Chinese.

f Article translated from French.

g No. of controls in calculation of RR=1598.

h Measurement of food intake assessed for validity.

i RR presented for various types of meat combined to reflect total meat consumption.

for references), 16 of which also contained relative risk estimates associated with total fat intake. Nested case–control studies were treated as cohort studies for these analyses. If study results were presented in more than one article, the most recent analysis was used.

### Extraction and classification of data

Descriptive data regarding the number and type of subjects, estimates of mean daily dietary fat intake, method of dietary assessment and the partitioning of intakes for the calculation of relative risks were extracted from each article along with an estimate of relative risk and its associated 95% confidence (CI) interval. In these studies, the intake of fat or fat-containing foods was usually partitioned into tertiles, quartiles or quintiles. The relative risk of breast cancer comparing the highest with the lowest category of intake was extracted from each study. Relative risks and CIs were calculated for three studies (Graham *et al*, 1982, 1991; Yuan *et al*, 1995) and confidence intervals were calculated for five studies (Hirayama, 1978; Kinlen, 1982; Levi *et al*, 1993; Landa *et al*, 1994; Toniolo *et al*, 1994) by cell frequencies shown in the data or standard error values (Fleiss, 1981), and are thus unadjusted for other variables.

If the risk of breast cancer associated with the dietary variables was expressed in more than one way, the estimate extracted from the study was the one that reflected the greatest degree of controlling for confounders (i.e. risk factors and/or energy). When both hospital and population controls were used for comparison separately, the results for population controls were chosen for analysis. As few studies provided complete data for pre- and postmenopausal women separately, we chose the relative risk for the whole group if available. In some reports unadjusted relative risks were given, accompanied by an explicit statement that the estimate was unchanged by adjustment for energy or other risk factors. In these cases, the relative risk given was regarded as having been adjusted.

In some instances, more than one estimate of risk were combined in order to increase the comparability of the studies. For example, in a number of studies of fat-containing foods, separate estimates of risk for red meat, poultry or pork consumption were reported. These separate risk estimates were combined into a total meat group by averaging the log of the risk estimates. CIs were calculated for the average relative risk using the variances of each separate risk estimate. In two studies, relative risk estimates were given for pre- and postmenopausal women separately (Pryor *et al*, 1989; Ambrosone *et al*, 1998) and in one study, risk estimates were given for pre- and postmarriage separately (Wakai *et al*, 2000). In each of these cases, the estimates were combined into one to represent all women in the manner described above. Similarly, in the cohort study reported by Hirayama (1978), relative risks given for meat intake divided by age category were combined to produce one risk estimate for the population.

### Methodological standards

A quality score was calculated for each study included in the meta-analysis. Four investigators (NFB, LJM, KNV and BSC) independently scored the studies based on predetermined methodological standards and any differences were resolved by discussion. The criteria included the provision of details on how the population had been assembled, whether histological confirmation of breast cancer had been obtained, the methods used to control for observer bias, a description of the method of measurement of nutrient and/or food intake, including data on validation and reproducibility and whether or not adjustment of risk estimates for potential confounding factors such as energy intake and traditional risk factors for breast cancer had been performed. Quality scores were not used to weigh the individual estimates of risk, but were used to divide the studies into groups for a stratified analysis based on quality score.

### Statistical methods and analysis

Studies were classified as case–control or cohort and statistical analyses were performed for each study design separately as well as for all studies combined. Analyses were also performed on subgroups of studies based on quality score, geographical area, type of control population and other study characteristics.

Statistical analyses were performed using SAS (SAS Institute, Inc., Cary, NC, USA) software and graphical displays of the results produced using S-PLUS (Insightful, Inc., Seattle, WA, USA) software. The data required by SAS for each study included the natural log of the adjusted odds ratios, and its 95% CI. From these, the program calculated the summary risk estimate and the associated standard error, which was used to determine the 95% CI.

Owing to diversity in the location, design and analysis of the various studies, we were aware that the true effects being estimated were likely to vary among studies. There were two sources of variability that had to be addressed: the usual sampling variation in the estimates and variation in the underlying parameter. To account for both sources of variation in this meta-analysis, we used the method of DerSimonian and Laird (1986), employing the SAS MIXED procedure in which the magnitude of the heterogeneity is estimated, and accounted for by assigning a greater variability to the estimate of the overall effect. Thus, we did not assume that the studies represented the same effect. Rather, the effects came from some statistical distribution of effects. The random effects model does not rely on homogeneity; on the contrary, it assumes heterogeneity. We also employed additional subgroup and regression analyses to try to account for the observed differences between studies, and to examine the potential influence of study design and execution, study population, geographical location, adjustment variables, partitioning cut points and methods of analysis.

## RESULTS

### Characteristics of studies reported

A total of 45 published studies, containing 46 estimates of risk, examined the role of dietary fat in relation to breast cancer risk by an analysis of nutrient intake. Of these, 31 were case control and 14 were cohort in design, and they contained a total of 25 015 cases of breast cancer and over 580 000 control or comparison subjects. Table 1 summarises selected characteristics of the published studies that examined the role of dietary fat in relation to breast cancer risk through an analysis of nutrient intake. In all, 22 studies were carried out in European countries (including Russia), five in Asian countries, and 15 in North America. In addition, two studies were conducted in Australia and one in Uruguay.

The studies included in Table 1 had varied methods of execution and analysis. A total of 27 studies used population-based comparison or controls, 12 selected comparison subjects from hospital or clinics, two studies selected comparison subjects from both these sources and four selected controls from other defined populations (i.e. sisters, neighbourhood, or centre). In total, 32 studies obtained dietary data using food frequency questionnaires, 12 with diet histories, one with a 24-h diet recall and one with food records and food frequency questionnaire. Food frequency questionnaires were sometimes administered by interview, and sometimes self-administered, and differed substantially in the number of food items included (data not shown in the table).

All the studies included in Table 1 analysed the relationship between breast cancer risk and nutrient intake by partitioning intake, 13 by quintiles, 21 by quartiles, seven by tertiles and one at the median. One study used deciles of intake and two used specific increments in fat intake. A total of 26 studies met at least six of the methodological standards that were applied, 16 met four or five standards and three met fewer than four standards.

### Estimates of risk for nutrient consumption

Figure 1

**Figure 1:**
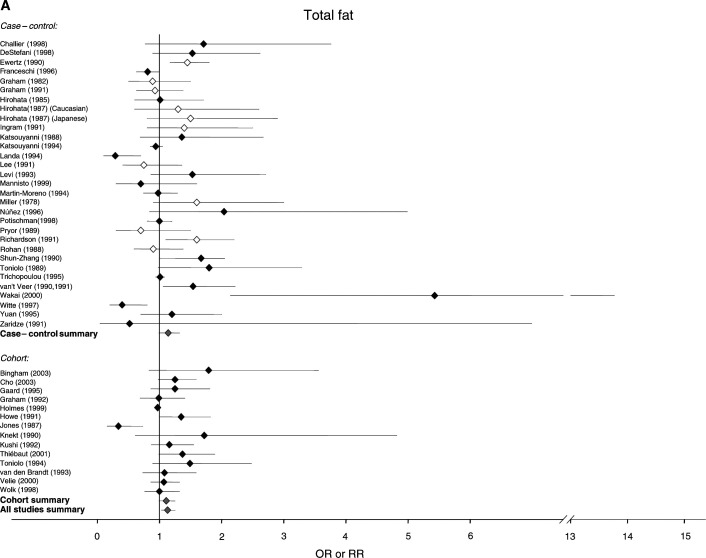

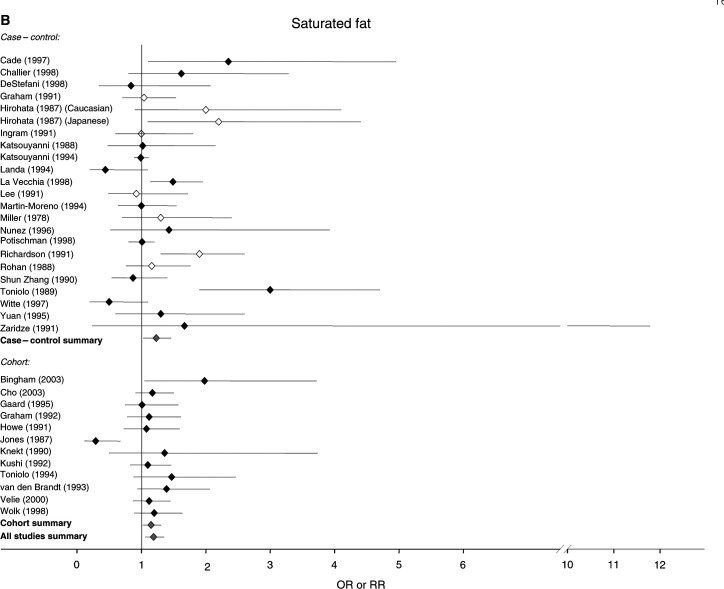

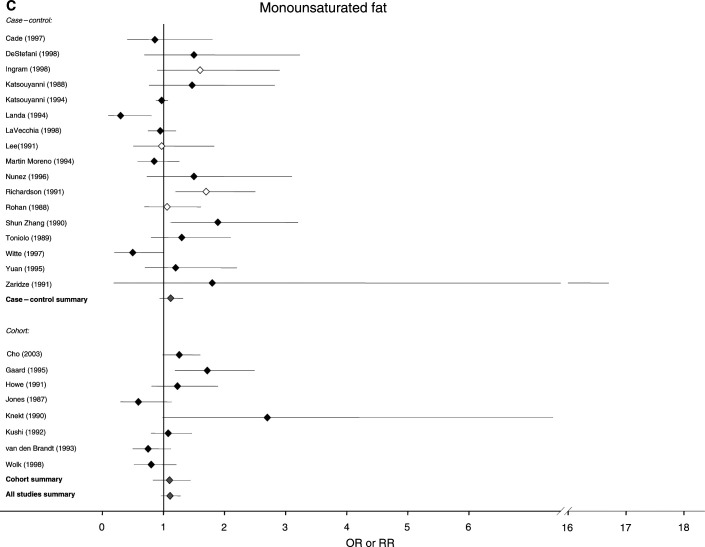

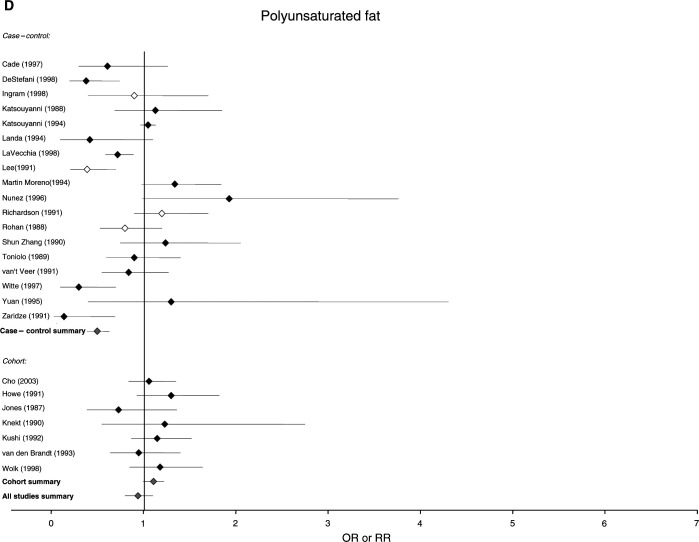
Relative risks for (**A**) total fat (**B**) saturated fat (**C**) monounsaturated fat and (**D**) polyunsaturated fat intake and breast cancer risk. CIs are 95%. Closed diamond=relative risk adjusted for energy intake. Open diamond=relative risk unadjusted for energy intake. Grey diamond=summary relative risk results of the meta-analysis.

shows the estimates of the risk of breast cancer generated by these studies for total fat, as well as saturated, monounsaturated and polyunsaturated fat, and indicates where risk estimates have been adjusted for energy intake and for established breast cancer risk factors. For total fat, the summary relative risk for all 46 estimates was 1.13 (95% CI: 1.03–1.25). Cohort studies had a summary relative risk of 1.11 (95% CI: 0.99–1.25) and case–control studies had a relative risk of 1.14 (95% CI: 0.99–1.32). Summary relative risks for both cohort and case–control studies that adjusted for energy intake and traditional risk factors for breast cancer were 1.13 (95% CI: 1.04–1.23) and 1.22 (95% CI: 0.91–1.63), respectively. The summary relative risks for saturated fat were greater than unity for all studies combined (RR, 1.19; 95% CI: 1.06–1.35), case–control studies alone (RR, 1.23; 95% CI: 1.03–1.46) and cohort studies alone (RR, 1.15; 95% CI: 1.02–1.30). The summary relative risk for monounsaturated fat was 1.11 (95% CI: 0.96–1.28) for all studies, 1.12 for case–control studies alone (95% CI: 0.94–1.32) and 1.10 for cohort studies alone (95% CI: 0.83–1.44). The summary relative risks for polyunsaturated fats were below unity for all studies and case–control studies alone (all studies, 0.94; 95% CI: 0.80–1.10, case control, 0.50; 95% CI: 0.39–0.63), but above unity for cohort studies alone (1.11; 95% CI: 1.00–1.22).

### Replication of published results of a combined analysis of cohort studies

To determine whether the methods used in the present paper could replicate those based upon an analysis using the data from individual studies, we applied our methods to a group of studies that were the subject of a previously published pooled analysis of seven cohort studies by Hunter *et al* (1996). For our analysis, we extracted risk estimates and 95% CIs from the original papers and calculated the summary risk estimates as described above. Estimates for total fat were available for five of the seven studies analysed by Hunter *et al* (Howe *et al*, 1991a; Graham *et al*, 1992; Kushi *et al*, 1992; Willett *et al*, 1992; van den Brandt *et al*, 1993). Comparing our results with those of Hunter's analysis, the summary relative risks for total fat were, respectively, 1.06 (95% CI: 0.92–1.23) and 1.05 (95% CI: 0.94–1.16), for saturated fat 1.05 (95% CI: 0.90–1.23) and 1.07 (95% CI: 0.95–1.20), for monounsaturated fat 0.96 (95% CI: 0.83–1.10) and 1.01 (95% CI: 0.88–1.16) and for polyunsaturated fat 1.14 (95% CI: 0.98–1.34) and 1.07 (95% CI: 0.97–1.17), respectively. Our calculations thus produced risk estimates and CIs very similar to those reported from the pooled analysis.

### Characteristics of studies reporting analysis according to foods

**Figure 2:**
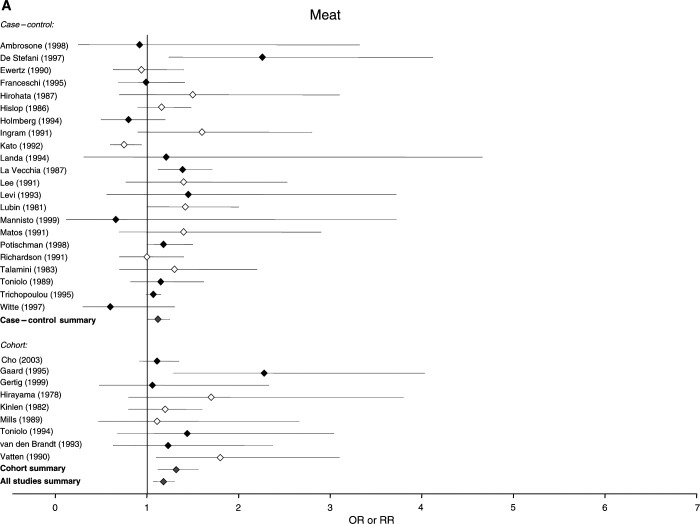

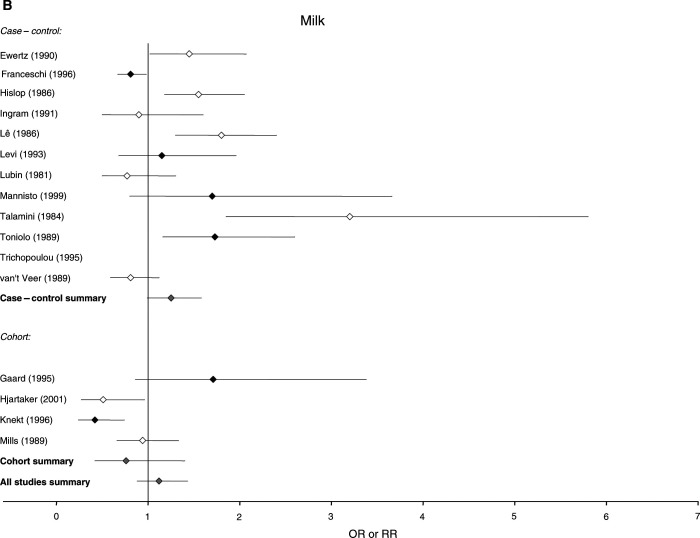

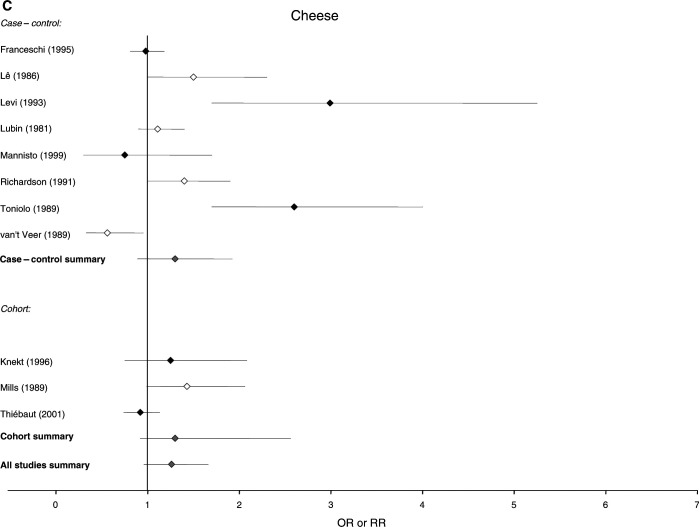
Relative risks for (**A**) meat (**B**) milk and (**C**) cheese intake and breast cancer risk. CIs are 95%. Closed diamond=relative risk adjusted for energy intake. Open diamond=relative risk unadjusted for energy intake. Grey diamond=summary relative risk results of the meta-analysis.

The 37 studies that examined food consumption in relation to breast cancer risk, 25 case–control and 12 cohort in design, included a total of 20 571 cases and over 490 000 control or comparison subjects. The 37 studies contained 31 estimates of risk for meat, 16 for milk and 11 for cheese. There is some overlap, as 16 studies reported risk in relation to consumption of both nutrients and foods, and are therefore included in both Figures 1 and 2

.

Table 2 summarises selected characteristics of the published studies that examined the role of diet in relation to breast cancer risk by an analysis of food intake. A total of 20 studies were carried out in European countries, 10 in North America, four in Asian countries and one in each of Argentina, Australia and Uruguay. A total of 24 studies used population-based comparison or controls, 10 selected comparisons from hospitals and three selected comparisons from other populations (i.e. neighbourhood and hospital visitors). All but seven studies obtained dietary data using a food frequency questionnaire, of which two used unspecified methods.

All the studies included in Table 2 analysed the relationship between breast cancer risk and food intake by partitioning intake. Differences in the methods of partitioning existed not only between studies but also within studies analysing intake of different foods. In all, 13 studies met at least six of the methodological standards that were applied, 18 met four or five, and six met fewer than four standards.

### Estimates of risk for food consumption

Figure 2 shows the distribution of the estimates of risk of breast cancer and the 95% CIs generated by the studies for intake of meat, milk and cheese. The summary relative risks for meat intake were 1.17 (95% CI: 1.06–1.29) for all studies, 1.13 (95% CI: 1.01–1.25) for case–control studies alone and 1.32 (95% CI: 1.12–1.56) for cohort studies alone. The summary relative risks for milk were 1.12 (95% CI: 0.88–1.43) for all studies, 1.25 (0.99–1.58) for case–control studies alone and 0.76 (95% CI: 0.42–1.40) for cohort studies alone, and the summary relative risks for cheese were 1.26 (95% CI: 0.96–1.66) for all studies and 1.30 (95% CI: 0.89–1.92) for case–control studies alone.

### Analysis of sources of variation for studies of total fat and breast cancer risk

As has already been noted, the studies included in the analysis differed in a number of aspects of their design and execution, and were reported from countries that are known to have wide differences in breast cancer risk. We examine below the influence of some of these sources of heterogeneity on the results presented in the previous sections. Owing to the small number of studies available after division into subgroups, we have confined our attention to those studies that reported the results of nutrient analysis for total fat intake and breast cancer risk.

The principal sources of variation in the study methodology examined were the extent to which studies met the methodological standards described above, the sources from which control or comparison groups were selected, the partitioning of nutrient intake and the geographic region where the studies were carried out.

#### Methodological standards

The summary relative risks were calculated for studies classified according to the proportion of methodological standards met (see Methods section). The summary relative risk for the relationship of total fat intake to breast cancer risk, for all 26 studies that met 80% or more of the standards, was 1.17 (95% CI: 1.03–1.32). For the 11 studies that met between 70 and 80% of standards, the summary relative risk was 1.08 (95% CI: 0.93–1.24), and for the nine studies that met 70% or less of the standards the relative risk was 0.91 (95% CI: 0.59–1.40).

#### Source of controls

The summary relative risk for total fat and breast cancer risk was 1.14 (95% CI: 1.04–1.25) for the 25 studies in Figure 1 that selected control or comparison groups from defined nonhospital populations. The 11 case–control studies in this group had a summary relative risk of 1.12 (95% CI: 0.96–1.31). The 14 case–control studies that selected controls from hospital populations had a summary relative risk of 1.11 (95% CI: 0.84–1.47).

#### Partitioning of nutrient intake

The summary relative risk for studies that partitioned nutrient intake into quintiles was 1.07 (95% CI: 0.94–1.21) for all studies and 1.01 (95% CI: 0.83–1.24) for case–control studies; for studies that used quartiles, 1.12 (95% CI: 0.95–1.32) for all studies and 1.16 (95% CI: 0.91–1.48) for case–control studies; and for studies that used tertiles, 1.15 (95% CI: 0.66–1.99) for all studies and 1.07 (95% CI: 0.56–2.05) for case–control studies.

#### Geographic variation

To examine the possible influence of the country in which they were carried out, studies were divided into four geographical categories. The summary relative risk for European studies (*n*=22) was 1.17 (95% CI: 1.02–1.34); for North American studies (*n*=15) 1.04 (95% CI: 0.91–1.18) and for Asia (*n*=6) 1.42 (95% CI: 0.87–2.30).

#### Regression analysis

To examine the independent contribution of the factors considered above, regression analysis was carried out, in which the log of the relative risk for total fat intake in each study, weighted by the reciprocal of its variance, was the dependent variable and study quality score, geographical area, study design and type of controls were the independent variables. However, univariate analysis showed none of these variables to be significantly associated with the response; but, the type of controls and geographic location were significantly associated with the log-relative risk when they were both in the model. Studies using population-based controls had higher relative risks than those using hospital-based controls (*P*=0.002), and both European and Asian studies had higher relative risks than North American studies (*P*=0.006 and 0.05, respectively). Interactions between all four variables were examined and no significant interactions were found.

## DISCUSSION

This quantitative summary of the published literature on the risk of breast cancer associated with dietary fat intake suggests that a higher intake of fat is associated with an increased risk of breast cancer. The summary relative risk for all studies that examined nutrient intake is calculated from the results of cohort and case–control studies, and in contrast to our previous publication, the results from these different designs for epidemiological investigation gave very similar results. This conclusion is based on 45 studies that contain a total of 25 015 cases of breast cancer and 580 000 control or comparison subjects. The summary risk estimates from all case–control and cohort studies were very similar, although neither was statistically significant. The combined estimate, however, was statistically significant as was the summary risk estimate for cohort studies that met 80% or more of the quality standards.

Other differences between our earlier analysis and the present findings are summarised in Table 3

**Table 3:** Summary risks for 1993 and present meta-analyses

**Fat/food type**	**Variable**	**1993 revised analysis**	**Present analysis**
*Total fat*	*Number of studies*		
	Case–control	16	31
	Cohort	7	14
	Combined	23	45
	*All studies*		
	Case–control	1.26 (1.10–1.45)	1.14 (0.99–1.32)
	Cohort	1.02 (0.80–1.31)	1.11 (0.99–1.25)
	Combined	1.17 (1.03–1.32)	1.13 (1.03–1.25)
	*High quality*		
	Case–control	1.45 (1.15–1.84), *N*=5	1.22 (0.91–1.63), *N*=13
	Cohort	1.07 (0.93–1.24), *N*=6	1.13 (1.04–1.23), *N*=13
	Combined	1.23 (1.06–1.43), *N*=11	1.17 (1.03–1.32), *N*=26
	Country		
	North America	1.03 (0.85–1.24), *N*=10	1.04 (0.91–1.18), *N*=14
	Europe	1.44 (1.30–1.60), *N*=8	1.17 (1.02–1.34), *N*=21
	Asia	—	1.42 (0.87–2.30), *N*=6
	Other	1.13 (0.84–1.51), *N*=5	1.20 (0.93–1.56), *N*=4
			
*Types of fat*			
Saturated	Number of studies	11	22
	Summary risk, all studies	1.21 (0.98–1.49)	1.18 (1.04–1.34)
Monounsaturated	Number of studies	15	24
	Summary risk, all studies	1.19 (1.01–1.40)	1.10 (0.95–1.28)
Polyunsaturated	Number of studies	15	24
	Summary risk, all studies	0.97 (0.83–1.13)	0.92 (0.78–1.09)
			
*Food types*			
Meat	Number of studies	17	31
	Summary risk, all studies	1.20 (1.07–1.34)	1.17 (1.06–1.29)
Milk	Number of studies	10	16
	Summary risk, all studies	1.22 (0.91–1.64)	1.12 (0.88–1.43)
Cheese	Number of studies	6	11
	Summary risk, all studies	1.32 (0.90–1.93)	1.26 (0.96–1.66)

. (The software used for our earlier analysis contained a programming error, which had a small influence on the results, but did not affect the conclusions of the paper. The table shows the corrected values of the published results.) Compared to the 1993 analysis, which was based on 23 studies, the present analysis based on 45 studies, gave smaller odds ratios for case–control studies, and slightly larger relatives risks for cohort studies. Neither study design gave significant estimates of risk in the previous or present analysis, but the combined estimates were significant in both. Among studies of higher quality, the estimate from cohort studies was significant in the present results, while the estimate from case–control studies was no longer significant. Strong evidence of substantial variation in results according to the geographical location of the study was present in both analyses. Point estimates of risk associated with fat intake were highest in Asia, lowest in North America and intermediate in Europe, findings that may be related to differences in the underlying variation in dietary fat intake in the populations in these regions.

Different studies partitioned fat intake in different ways, but an examination of the results obtained suggested that partitioning by tertiles, quartiles or quintiles gave very similar estimates. Among the major subtypes of fat, we found that saturated fat was significantly associated with breast cancer risk in both case–control and cohort studies, and that results were significant in the present but not the previous analysis. Mono- and polyunsaturated fat were not significantly associated with breast cancer in either case–control or cohort studies, or in summaries of all studies in the present analysis.

Our conclusion about the relationship of dietary fat to risk of breast cancer is supported to some degree by studies of specific foods. Of the studies that examined intake of foods in relation to risk of breast cancer, the largest number had examined meat consumption, which was significantly associated with breast cancer risk in this meta-analysis, in the overall estimate of risk and in both case–control and cohort studies considered separately. Fewer studies examined milk and cheese intake in relation to breast cancer risk, and although point estimates for the summary relative risks of all studies were greater than unity for both foods, neither was statistically significant.

Although this meta-analysis was based on published results, we were able to generate results similar to those of a previously published combined analysis of a subset of the cohort studies examined here. The differences between the results obtained in case–control and cohort studies might be attributable to recall bias, but as similar results were found here in the two research designs it is not likely that this potential source of bias has a major influence.

The biological plausibility of an association between dietary fat and breast cancer risk is shown by the effect that dietary fat intake has on mammary carcinogenesis in animals (see, for reviews, Freedman *et al*, 1990; Welsch, 1994),which appears to be distinct from the effect of calories, as well as by the known biological effects of fat. Potential mechanisms include the generation from fatty acids of eicosanoids, the generation of free radicals and mutagenic compounds such as malondialdehyde by lipid peroxidation and the modulation of genes that are involved in mammary carcinogenesis (Cohen *et al*, 1986).

Despite the strong evidence that breast cancer is influenced by environmental factors, and the consistency of the ecological analyses suggesting that dietary fat is one of these factors, epidemiological investigations of the relationship of dietary fat to breast cancer incidence based upon the measurement of dietary intakes in individuals with case–control and cohort studies, have given much less consistent results. However, in considering these results, and those given above in our quantitative summary of the published literature, we need to consider the effects of the relative homogeneity of fat intake within populations and error in the measurement of fat intake, both factors that are expected to attenuate any true association between dietary fat and breast cancer.

For example, homogeneity is shown by the range across quintiles of total fat intake in the Nurses Health Study (Willett *et al*, 1987), a large cohort study in North America, which was only 32–44% of calories, compared to the international range of 15% or less to more than 40% of calories. This narrow range of fat intake is expected, from international data, to be associated with a relative risk of only 1.4 in the highest quintile of fat intake relative to the lowest. When the measurement error known to be associated with the food frequency questionnaire used is taken into account, this estimate of the relative risk is reduced to 1.16, a figure that is close to the summary relative risk of our meta-analysis (Prentice *et al*, 1988).

Measurement error in the food frequency questionnaires used in most studies may lead to overestimation of the range of intakes and may also lead to attenuation of risk (Prentice, 2003). The cohort study of Bingham *et al* (2003) showed a small and nonsignificant increase in the risk of breast cancer when fat intake was estimated from a food frequency questionnaire, but a larger and significant increase when estimated from food records obtained from the same subjects.

Experimental trials, in which the range of fat intake is increased beyond that seen in most Western populations, are a means of overcoming the limitations of observational epidemiology that arise from homogeneity of intake and measurement error, and provide the strongest evidence available concerning a causal relationship of dietary fat intake to breast cancer risk. Further, such trials are the only means available to determine whether breast cancer risk in high-risk subjects can be reduced by changing dietary fat intake.
